# TumorTwin: a Python framework for patient-specific digital twins in oncology

**DOI:** 10.1186/s12911-026-03520-2

**Published:** 2026-05-11

**Authors:** Michael G. Kapteyn, Anirban Chaudhuri, Ernesto A. B. F. Lima, Graham Pash, Rafael Bravo, Karen E. Willcox, Thomas E. Yankeelov, David A. Hormuth II

**Affiliations:** 1 Oden Institute for Computational Engineering and Sciences, The University of Texas at Austin, Austin, TX USA; 2 Department of Biomedical Engineering, The University of Texas at Austin, Austin, TX USA; 3 Texas Advanced Computing Center, The University of Texas at Austin, Austin, TX USA; 4 Department of Diagnostic Medicine, The University of Texas at Austin, Austin, TX USA; 5 UT Austin Cancer Research Center, The University of Texas at Austin, Austin, TX USA; 6 Department of Imaging Physics, MD Anderson Cancer Center, Houston, TX USA

**Keywords:** Digital twin, Python, Differentiable programming, Computational oncology, Image-based modeling, Magnetic resonance imaging, Software

## Abstract

**Background:**

Advances in the theory and methods of computational oncology have enabled accurate characterization and prediction of tumor growth and treatment response on a patient-specific basis. This capability can be integrated into a digital twin framework in which bi-directional data-flow between the physical tumor and the digital tumor facilitate dynamic model re-calibration, uncertainty quantification, and clinical decision-support via recommendation of optimal therapeutic interventions. However, many digital twin frameworks rely on bespoke implementations tailored to each disease site, modeling choice, and algorithmic implementation.

**Results:**

We present TumorTwin, a modular and differentiable software framework for initializing, updating, and leveraging patient-specific cancer tumor digital twins. TumorTwin is publicly available as a Python package, with associated documentation, datasets, and tutorials. Novel contributions include the development of a patient-data structure adaptable to different disease sites, a modular architecture to enable the composition of different data, model, solver, and optimization objects, and CPU or GPU parallelized implementations of forward model solves and gradient computations. We demonstrate the functionality of TumorTwin via an in silico dataset of high-grade glioma growth and response to radiation therapy.

**Conclusion:**

The TumorTwin framework enables rapid prototyping and testing of image-guided oncology digital twins. This allows researchers to systematically investigate different models, algorithms, disease sites, or treatment decisions while leveraging robust numerical and computational infrastructure.

**Supplementary Information:**

The online version contains supplementary material available at 10.1186/s12911-026-03520-2.

## Background

The recent advancement of digital twin (DT) technologies for biomedical applications [28, 40, 43, 48], in general, and oncology [6, 49, 60], in particular, has the potential to transform personalized medicine by enabling accurate predictions of disease progression and treatment response as well as identifying improved therapeutic interventions [29, 61]. However, many DTs use custom numerical or computational methods, require proprietary code bases, and are tailored for each specific application resulting in limited portability across disease sites and application domains. While we advocate that DTs should be fit-for-purpose and tailored to their domain of application, there is substantial overlap in the data processing, modeling, and numerical solver infrastructure that can be generalized across disease sites to improve accessibility of DT technologies. To this end, we have developed an open-source software package that implements a DT framework with numerical and computational methods designed to facilitate the research and development of DT formulations in oncology.

At the core of a predictive DT in oncology is the computational model that represents a specific patient’s tumor and is used to forecast disease progression and response to therapy. However, given the complex multidisciplinary and multiscale nature of cancer, tumor growth modeling is an active area of research. These models often combine first-principles biology and physics with data-driven or phenomenological modeling. Many open questions remain about specific modeling choices, such as *which* effects to model, *how* to model these effects, and how these choices *impact* model accuracy and computational cost [26, 33]. Additionally, using medical imaging data to calibrate DT models requires complex data processing pipelines (e.g., image registration and segmentation), and the impact of these algorithms on model quality is under-examined [3, 24]. Furthermore, formulation and numerical implementation of calibration and solution algorithms can impact the stability and quality of model predictions [12]. A thorough exploration of these tradeoffs requires a modular and adaptable modeling framework so that researchers can establish a baseline end-to-end DT architecture, and then easily experiment with various data, modeling, and algorithmic choices.

Recognizing the need for an adaptable framework to support research in DTs for oncology, we have developed TumorTwin, a Python framework for image-guided tumor modeling that supports different data sources, tumor growth models, treatment modules, model parameterizations, numerical solvers, and numerical optimizers. As a baseline, we provide a DT architecture consisting of a well-established tumor growth and treatment model, a suite of performant numerical solvers that support efficient gradient computation, and a collection of numerical optimizers for deterministic model calibration within a modular, differentiable programming framework. While there exist other frameworks that facilitate computational modeling of cancer, (eg. HAL [4], Chaste [39], Physicell [11], Netlogo [55], CellSys [1], Compucell 3D [54], and Morpheus [50]), our framework is the first that is specifically designed as an end-to-end (i.e., data-to-decisions) DT framework leveraging tissue-scale imaging data. In particular, creating a DT with a modeling-only library would require the user to implement separate algorithms for data processing and model calibration; we provide a complete high-performance pipeline for this purpose. We demonstrate the use of this framework for creating a DT of a high-grade glioma (HGG) [22] with a second demonstration focused on triple negative breast cancer [24] provided as Supplementary Material. For both cases, the DT is initialized for an individual patient using their quantitative magnetic resonance imaging (MRI) data to generate a personalized tumor growth model [17, 24]. Future MRI visits can be used to further calibrate model parameters related to tumor growth and response to treatment. The calibrated DT can be used to predict growth under different candidate treatments, which in turn can be used to optimize treatment on a patient-specific basis.

This paper presents the TumorTwin Python package. We first provide a brief summary of the underlying mathematical model for tumor growth and response to treatment, referring the reader to relevant literature that has developed this theory. We then discuss design principles, and implementation details of the TumorTwin package. Finally, we present a demonstrative case-study using an in silico HGG patient, intended to showcase the package functionality and provide a performance analysis for a representative use-case.

## Methods

This section briefly summarizes a class of mathematical models, formulated here as differential equations, for tumor growth and treatment response that underlies the TumorTwin software package. We also describe the model calibration process that makes these baseline models patient-specific. We emphasize that the focus of the current paper is on presenting a user-friendly software package that enables high-performance implementation of this class of model, rather than further developing the model itself. To this end, we consider a model of tumor growth and treatment response in the form of a system of ordinary differential equations, such as those those arising from the spatial discretization of a time-evolving PDE [46], in the form:

1 $$ \frac{du}{dt} = f(u,t,\boldsymbol{p}) $$

2 $$u(0) = u_0$$

where $u(t)$ represents some characteristic of the tumor, $t$ represents time, $u_0$ is the initial condition, and $\boldsymbol{p}$ represents a set of model parameters. This is the most general form of model that can be implemented in TumorTwin.

On top of this general foundation, TumorTwin also provides a specific implementation of a commonly used mathematical model [18, 22, 24], which takes the form of a reaction-diffusion partial differential equation (PDE) with chemotherapy (CT) and radiotherapy (RT) treatment effects. The reaction-diffusion model describes the change in the normalized tumor density due to tumor cell invasion (diffusion), logistic growth (reaction), and death due to treatment (RT and CT):

3 $$\begin{aligned} \frac{\partial N(\boldsymbol{x},t)}{\partial t} &= \underbrace{\nabla \cdot \left(D \nabla N(\boldsymbol{x},t) \right)}_{\mathrm{invasion}} \notag + \underbrace{k(\boldsymbol{x})N(\boldsymbol{x},t) \left(1 - \frac{N(\boldsymbol{x},t)}{\theta} \right)}_{\text{logistic growth}} \notag \\ &\quad \underbrace{- \sum_{i=1}^{n_{\mathrm{CT}}} \sum_{j=1}^{T_i}\alpha_i C_i \exp\left(-\beta_i\left(t-\tau_{i,j}\right)\right) N(\boldsymbol{x},t)}_{\mathrm{chemotherapy}},\end{aligned}$$

4 $$N(\boldsymbol{x},t)_{\mathrm{after}} = \underbrace{N(\boldsymbol{x},t)_{\mathrm{before}} \exp\left(-\alpha_\mathrm{RT}d_\mathrm{RT}(t)-\beta_\mathrm{RT}d_\mathrm{RT}^2(t)\right)}_{\mathrm{radiotherapy}}$$

where $N$ is the normalized tumor cell density (units: unitless), $D$ is the tumor cell diffusion coefficient (units: $\mathrm{mm}^2/\mathrm{day}$), $k(\boldsymbol{x})$ is the tumor cell proliferation rate (units: $\mathrm{day}^{-1}$), $\theta$ is the carrying capacity (units: unitless, upperlimit of 1), $n_{\mathrm{CT}}$ is the number of different CT agents, $T_i$ is the total number of doses delivered for agent $i$, $\alpha_i$ is the efficacy of CT agent $i$ (units: $\mathrm{day}^{-1}$), $C_i$ is the normalized dose of the CT agent $i$, $\beta_i$ is the decay rate for CT agent *i* (units: $\mathrm{day}^{-1}$), and $\tau_{i,j}$ is the time of $j$-th administration of CT agent $i$. Equation (4) defines the effect of radiotherapy and is modeled as an instantaneous reduction in $N$ at the time of delivery, with the survival fraction based on the linear quadratic model [37]. Here $N_{\mathrm{before}}$ and $N_{\mathrm{after}}$ are the normalized tumor density immediately before and after an RT event, $\alpha_\mathrm{RT}$ and $\beta_\mathrm{RT}$ are radiosensitivity parameters (units: $\mathrm{Gy}^{-1}$ and $\mathrm{Gy}^{-2}$, respectively), and $d_\mathrm{RT}(t)$ is the RT dose delivered at time $t$. As a reference, Table 2 in Supplementary Material A summarizes model parameters and variables with their definition and units.

The model represented by Eq. (3) is spatially discretized using a finite-difference scheme [30], with a grid size that corresponds to the voxel size in the input MRI data. This gives rise to a system of coupled ODEs of the form Eq. (1) with discrete radiotherapy events:

5 $$ \frac{d\mathbf{N}}{dt} = D \mathbb{L} \mathbf{N} + k \mathbf{N} \left(1 - \frac{\mathbf{N}}{\theta} \right) - \sum\limits^{n_{\mathrm{CT}}}_{i=1} \sum\limits^{T_i}_{j=1} \alpha_i C_i \exp\left(-\beta_i\left(t-\tau_{i,j}\right)\right) \mathbf{N},$$

6 $$\mathbf{N}_{\mathrm{after}} = \mathbf{N}_{\mathrm{before}}\exp\left(-\alpha_\mathrm{RT}d_\mathrm{RT}(t)-\beta_\mathrm{RT}d^2_\mathrm{RT}(t)\right)$$

where $\mathbf{N}$ is vector in which each entry corresponds to the normalized tumor density in a particular voxel and $\mathbb{L}$ is the Laplace operator after discretization, e.g. *via* a second-order central difference scheme. Note that in general $k$ and $D$ may be spatial fields, but we here assume that they are homogeneous in the domain (i.e., $k$ and $D$ are scalars), which allows us to pre-assemble a Laplacian operator independent of $D$. The model parameters are $\boldsymbol{p} = \{k, D, \theta, \boldsymbol{\alpha}, \boldsymbol{\beta}, \alpha_\mathrm{RT}, \beta_\mathrm{RT}\}$, where $\boldsymbol{\alpha}=\left[\alpha_1,\dots,\alpha_{n_\mathrm{CT}}\right]$ and $\boldsymbol{\beta}=\left[\beta_1,\dots,\beta_{n_\mathrm{CT}}\right]$.

Solving this model on a patient-specific basis requires an initial tumor state, $\mathbf{N}(0)$, and patient-specific model parameters. We derive these from patient-specific functional imaging, in particular, we adopt an approach based on the apparent diffusion coefficient (ADC), which can be derived from diffusion-weighted MRI as discussed in previous studies [17, 24]. The $ADC$ is used to assign the observational data $\boldsymbol{o}=\left[o(t_1)^\top,\dots,o(t_{n_\mathrm{visit}})^\top \right]$ for $n_\mathrm{visit}$ number of patient imaging visits. The observation at each visit $\left\{t_i\right\}_{i=1}^{n_\mathrm{visit}}$ is defined as

7 $$ o(t_i) = N(\boldsymbol{x},t_i) = \frac{ADC_\mathrm{w}-ADC(\boldsymbol{x},t_i)}{ADC(\boldsymbol{x},t_i)-ADC_\mathrm{min}},$$

where $N(\boldsymbol{x},t_i)$ is the normalized tumor density at 3D position $\boldsymbol{x}$ and time $t_i$, $ADC_\mathrm{w}$ is the $ADC$ of water at room temperature ($3.0 \times 10^{-3}\ \mathrm{mm}^2/\mathrm{s}$ [57]), $ADC_\mathrm{min}$ is defined as the minimum observed $ADC$ within the tumor region of interest. To maintain consistent values throughout simulation this is set to $0.0 \times 10^{-3}\ \mathrm{mm}^2/\mathrm{s}$. However, for large cohort studies, this value could be calculated using the minimum value for $ADC$ observed within the cellular dense tumor region of interest to get a more accurate reflection of the range of expected $ADC$ for disease type and diffusion protocol. The ADC_to_cellularity function only assigns $N$ within the tumor regions of interest, and assigned zero-elsewhere. $N(\boldsymbol{x},t_i)$ is bounded between 0 and 1, values outside of this range are set to the upper or lower bounds, respectively. We note, that while there have been several studies demonstrating ADC correlates with tumor cell density [2, 44, 52], there are numerous factors (e.g., extracellular space tortuosity, cell size, membrane permeability, and tissue temperature) which can influence the accuracy of these estimates.

The model is initialized using the observational data from the first patient visit to compute an initial condition $u_0 = \mathbf{N}_0 = o(t_1)$ using Eq. (7). The remaining data, $o_2,\ldots,o_{n_{visits}}$, can be used to calibrate the model parameters, $\boldsymbol{p}$, so that the model prediction matches the patient-specific tumor dynamics observed in the data. To do this, we solve an optimization problem of the form

8 $$ \boldsymbol{p}^* = \underset{\boldsymbol{p}\in\mathcal{P}}{\arg\min}\ \mathcal{L}(\boldsymbol{p}; \boldsymbol{o}),$$

where $\boldsymbol{p}^*\in \mathcal{P} \subseteq \mathbb{R}^{n_p}$ are the $n_p$ patient-specific parameters that best match the data $\boldsymbol{o}$. Here $\mathcal{L}$ is a user-defined scalar loss function that describes the distance between the model predictions and the patient-specific observations for a given value of $\boldsymbol{p}$. A common loss function that accounts for the spatial distribution of the tumor is the voxel-wise mean squared error between predicted cellularity maps and cellularity maps derived from observed ADC measurements.

Another scalar quantity of interest that reflects the combined size and intensity of the tumor is the total tumor cell count (TTC), which can be computed by multiplying $N$ by the maximum number of cells that can occupy a voxel, $\mathbf{\theta_{cells}}$, and summing across all voxels in the computational domain as

9 $$\mathrm{TTC}(t) = \sum_\mathbf{i} \mathbf{N}_i(t)\theta_{cells}.$$

Our TTC calculation assumes a fixed $\theta_{cells}$ based on an assumed packing packing fraction and average cell volume [24]. For more precise TTC calculations, TTC values could be normalized to a reference tissue with a known cell density.

## Implementation

### Software design

The goal of this software package is to empower researchers to develop high-performance predictive DTs for oncology, leveraging medical imaging datasets and incorporating treatment protocols. A key focus of our design is to balance performance with usability, ensuring that researchers can easily modify and extend various aspects of the data pipeline, computational models, and solvers. This allows researchers to explore new modeling directions and computational technologies in the context of an end-to-end DT workflow.

To achieve this flexibility, we have developed a modular codebase with well-defined abstractions. This modularity enables researchers to swap, customize, or extend different components without requiring deep modifications to the core framework. Each module is designed to interact seamlessly with others, facilitating an intuitive workflow for building and refining patient-specific DTs. This standardized workflow guides users through the key steps of building a predictive model:

1. Prepare input data (e.g., imaging, treatment history).
2. Construct a PatientData object to encapsulate all relevant patient-specific information.
3. Generate a TumorGrowthModel object based on this patient data, encoding tumor growth and treatment response dynamics.
4. Wrap the model in a Solver, which performs numerical integration or simulations to generate predictions.
5. Optionally use an Optimizer, which refines model parameters to best match observed data.

**Fig. 1:**
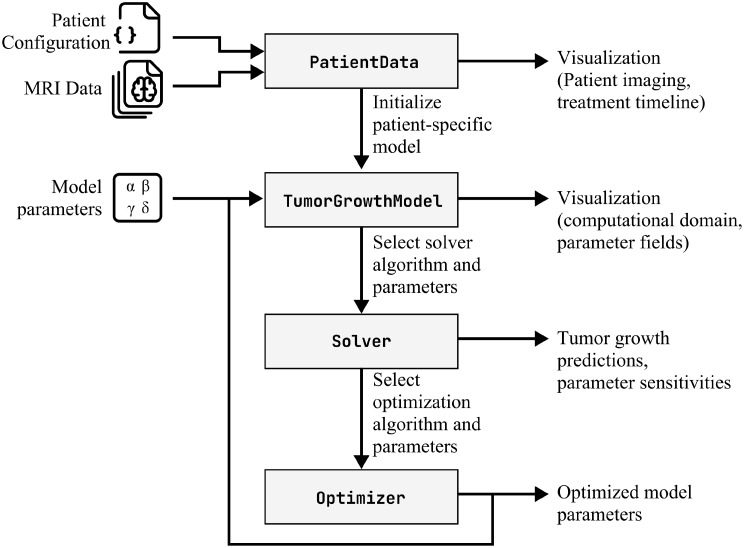
Tumortwin workflow key components. Tumortwin takes as inputs the patient configuration file, MRI data, and an initial guess of model parameters. The patient configuration file and MRI data are used to construct a patientdata object which serves as the central data object throughout tumortwin. The patientdata object is used to initialize a patient-specific tumorgrowthmodel, which can be combined with a forwardsolver to simulate tumor growth over time. If longitudinal data is available within the patientdata object, an optimizer module can be used to calibrate model parameters by minimizing the error between model predictions and patient-specific measurements

This workflow ensures clarity and reproducibility while allowing researchers to incorporate custom components at each stage. For example, the Model can be expanded to include new treatment terms or biological features (e.g., mechanics-coupled tumor growth [20]) Fig. 1 provides a high-level overview of the package structure, illustrating how these components interact. Each element is described in detail in the following sections.

The package is written entirely in Python and leverages the differentiable programming paradigm through PyTorch, providing several advantages:

1. GPU compatibility, allowing computationally intensive tumor simulations to run efficiently on either CPU- or GPU-based computing platforms.
2. Automatic differentiation, enabling sensitivity analysis and seamless integration of gradient-based optimization techniques for parameter fitting.
3. Extensibility, as users can compose our models with other PyTorch-based architectures, such as neural ODEs, learned pre-processing pipelines, or deep-learning-based post-processing steps.

### Example datasets provided with the package

The primary data source used in our framework is medical imaging data, which serves as the foundation for constructing and calibrating patient-specific DT models. However, working with medical imaging data presents several challenges, e.g., large file sizes and complex file formats with multiple conventions for coordinate systems, units, and other metadata. These factors make preprocessing and standardization critical steps in any DT pipeline.

In addition to these technical challenges, the use of real patient imaging data is further complicated by patient privacy concerns. Sharing clinical imaging datasets openly typically requires extensive de-identification procedures and complex institutional approval processes. As a result, publicly available datasets that can be used for testing and benchmarking are often limited in scope and accessibility.

To provide users with accessible demonstrations and a reference for dataset creation, we have developed and included two datasets that have been synthesized using real patient data as a reference:

1. A dataset for high-grade glioma (HGG), featuring synthetic brain MRI scans and corresponding radiotherapy and chemotherapy schedules.
2. A dataset for triple-negative breast cancer (TNBC), containing synthetic breast imaging data and chemotherapy schedules.

These datasets are designed to facilitate quick exploration of the software’s capabilities without requiring access to real clinical data, while also serving as structured templates for users who wish to integrate their own patient datasets into the framework. The procedure for generating these synthetic datasets is detailed in Supplementary Material B.

### Importing and pre-processing patient data

A critical first step in constructing a DT is importing patient-specific data, which typically includes medical imaging and treatment history. Our package is designed to handle these inputs efficiently while ensuring interoperability with existing medical imaging and data processing tools.

To facilitate integration with existing medical imaging workflows, our package supports the *Neuroimaging Informatics Technology Initiative* (NIfTI) format, a widely used format for medical imaging data. We provide a lightweight wrapper around the NIfTI classes from established Python libraries, including nibabel and ITK/SimpleITK. This approach ensures full interoperability with these widely adopted toolkits, allowing users to leverage their extensive functionality for image processing, registration, and analysis while using our package for DT modeling.

Ensuring the integrity and compatibility of patient data is essential for reliable model predictions. To this end, we use the Pydantic data validation library to define a structured data model with built-in validations. We implement a BasePatientData class, which defines a base data model comprised of one-off imaging data (e.g. anatomic masks), longitudinal treatment data, and a list of visits, each associated with a time and visit-specific imaging dataset. We implement specialized subclasses for different cancer types, e.g.,the HGGPatientData class specifies the imaging formats required, for a HGG DT. For the HGGPatientData class, standard anatomical ($T_1$-weighted and $T_2$-fluid attenuated inversion recovery (FLAIR)) and functional ($ADC$, from diffusion-weighted MRI) are required.

To provide flexibility in bundling imaging data across multiple modalities and multiple imaging visits, alongside patient treatment data, we employ JSON-based configuration files which are loaded into the corresponding pydantic patient data object. JSON is a lightweight and platform-agnostic format, which allows users to easily store, share, and version control their DT configuration files. When creating a patient data object (either manually via Python code, or by loading a JSON file), Pydantic validation verifies that the provided MRI data and treatment records are consistent and complete, helping to catch formatting issues early and reducing potential errors in model building and downstream model computations.

To assist in verifying and interpreting input data, we provide a visualization method for PatientData objects. This method generates a summary figure that includes:

- A treatment timeline, displaying administered therapies, doses, and imaging timepoints.
- The provided medical images and regions of interest.

**Fig. 2:**
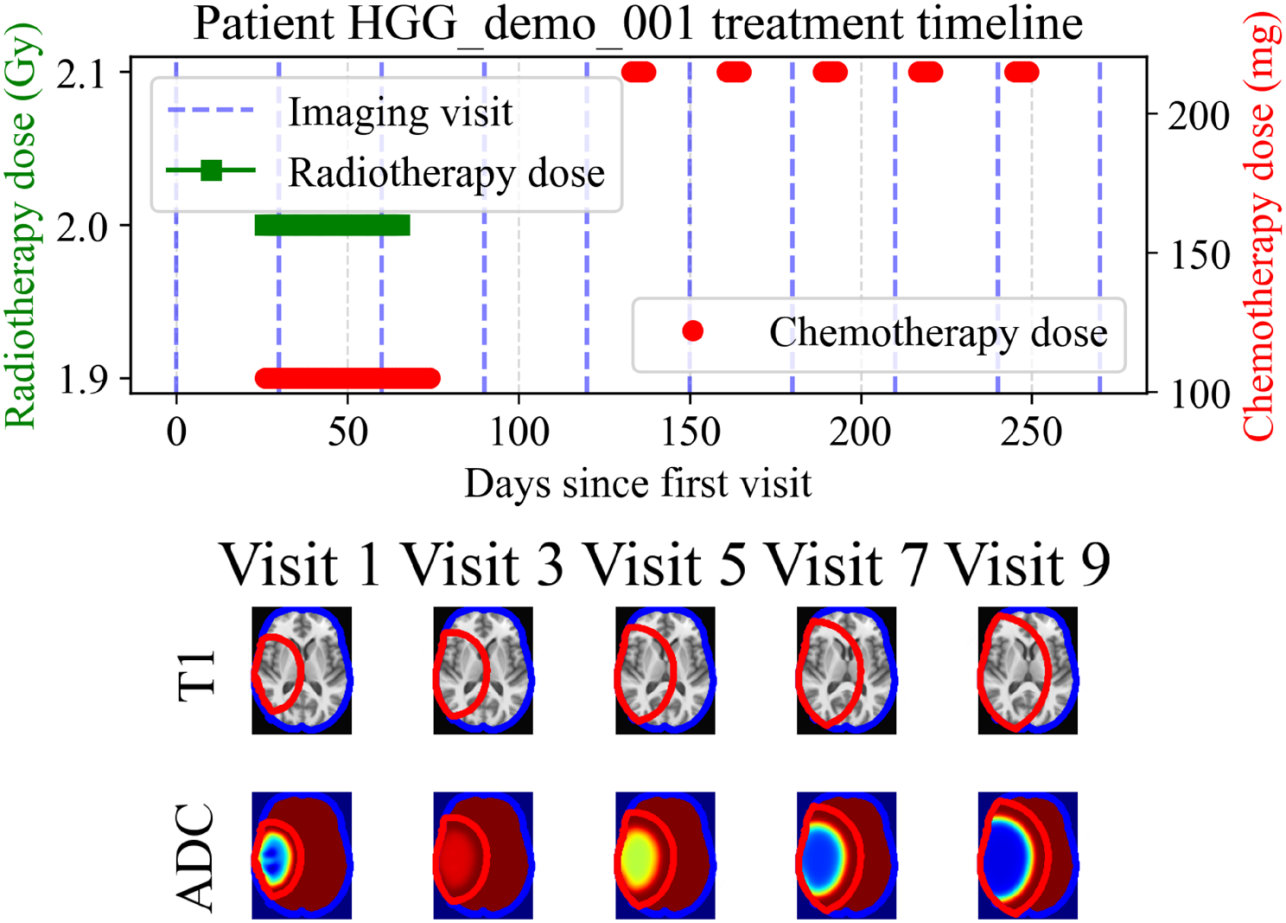
Patient data summary graphics. Example output of the patient data summary function applied to the in silico HGG dataset. This graphic shows treatment and imaging schedule (top panel), anatomical $T_1$-weighted MRI with associated tumor segmentations (middle panel), and the apparent diffusion coefficient ($ADC$) map for the same imaging slices (bottom panel). In practice, this summary can be used to visually confirm longitudinal registration of imaging series, accuracy of tumor segmentations, and treatment details

These visualizations serve as a quick diagnostic tool, allowing users to inspect patient-specific data before initiating model simulations as shown in Fig. 2.

### Creating a model

The base TumorGrowthModel class in TumorTwin provides a template for tumor growth models of the general form given by Eq. (1). The key required method in this class is the forward method, which evaluates the right-hand side of Eq. 1, using parameters and variables stored in the solver object. This allows users to implement their own mathematical model by implementing a subclass of the base tumor growth model complete with a corresponding forward method.

In addition to the base class, we provide an implementation of the reaction-diffusion tumor growth and treatment model (Eqs. (3)-(4)) in a subclass called ReactionDiffusion3D. Creating this model requires inputs like a PatientData object, and model parameters such as $k$, $D$, and $\theta$. It also implements RT and CT treatment effects via RadiotherapySpecification and ChemotherapySpecification inputs, respectively. Each treatment specification stores the relevant treatment dosage schedule and treatment model parameters.

The provided model is intended to serve as a flexible template, enabling the adaptation of existing models or the integration of new ones while ensuring compatibility with the Solver, PatientData, and Optimizer objects. This design facilitates the rapid deployment of novel models within the DT framework.

### Model prediction via high-performance solvers

Generating tumor growth predictions requires solving the coupled set of ODEs defined by a tumor growth model in the form of Eq. (1). As the size of the state vector $u$ can be large (e.g., equal to the number of imaging voxels in the computational domain for a spatially-discretized PDE model such as Eq. (5)) an efficient numerical integration scheme is crucial for tractable simulation. In this work, we employ the torchdiffeq library [7], which provides differentiable ODE solvers compatible with the PyTorch framework. Available solver schemes include standard fixed-step schemes such as fourth-order Runge-Kutta (rk4) [13], as well as adaptive-step methods, such as the fifth-order Runge-Kutta of Dormand-Prince-Shampine (dopri5) [10]. The package provides a Solver implementation called TorchDiffEqSolver that is based on the torchdiffeq library and supports specifying output timesteps independently from the solver timesteps, and the handling of discrete events (for example, to implement the radiotherapy model given by Eq. (4)). As the solver is compatible with PyTorch, forward solves can be run on a GPU architecture simply by setting device = torch.device(”cuda”).

For the results reported in this work, we used the fourth-order Runge–Kutta (rk4) method with a step size of 0.5 days for simulations of both the HGG and TNBC models. One can modify the solver options through TorchDiffEqSolverOptions. We also provide notebooks in the tutorials folder that can reproduce the results presented here.

### Efficient gradient computation

In addition to solving a model forward in time to predict tumor growth for a given set of model parameters, our package also supports the efficient computation of gradients. When the provided tumor growth model is differentiable and PyTorch compatible, derivatives of output quantities with respect to input parameters can be computed efficiently via reverse-mode automatic differentiation (backpropagation) using the standard pytorch syntax. However, tumor growth models typically require a large number of states (e.g. equal to the number of imaging voxels in the computational domain), and a large number of successive timesteps. Thus, the computational graph that needs to be maintained in order to leverage automatic differentiation often becomes prohibitively large, exceeding the available memory of most systems. An alternative is to use the adjoint method for computing gradients, which requires $\mathcal{O}(1)$ memory, at the cost of requiring a backwards-in-time solve (the adjoint pass) through the model.

We leverage the adjoint method implemented in the solver library torchdiffeq [25], which is available via a simple keyword argument (use_adjoint = True) in the solver interface. This capability allows one to compute gradients of any scalar function of the solver output, with respect to any of the model inputs. For example, one could run a forward solve over a period of $200$ days to compute $\mathbf{N}(200)$, post-process the solution to compute $\mathrm{TTC}(200)$ via Eq. (9), and then run a backward pass to compute $\frac{\partial{(TTC(200))}}{\partial k}$, i.e., the rate of change of the solution with respect to the proliferation rate parameter, $k$.

### Model calibration via gradient-based numerical optimization

Calibrating the model parameters against observed data requires solving the optimization problem given in Eq. (8). In this work, we use the mean squared error loss function. To solve this problem we provide an LMOptimizer implementation of the base Optimizer class which implements the Levenberg-Marquardt (LM) algorithm for optimization. LM has been frequently used for patient-specific parameter calibration of PDE models of cancer [17, 19]. In addition, recall that we are able to compute gradients of the loss function with respect to parameters, as described in the previous section. Thus, our solver is compatible with the built-in gradient-based Pytorch optimizer objects (from the widely used torch.optim library). Using these objects, the user can easily customize the loss function and optimization algorithm, further extending the modularity of the TumorTwin framework.

## Results

### Model calibration demonstration on synthetic data

**Fig. 3:**
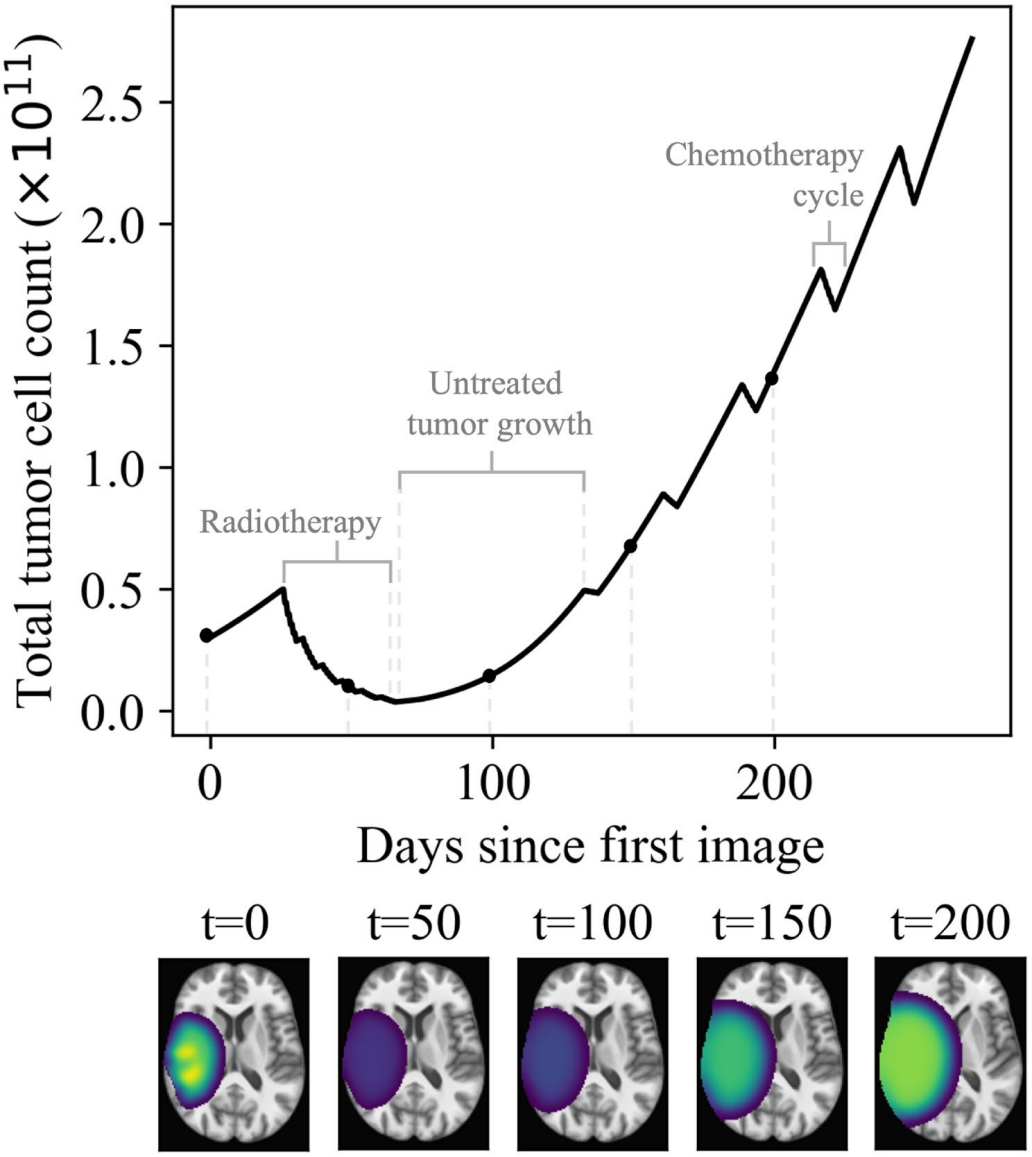
Example tumor growth prediction for the in silico HGG patient. A representative total tumor cell count prediction over time is shown (top panel), with 2D slices of the model solution at five snapshots in time (bottom panel). Note the invasion, logistic growth, chemotherapy, and radiotherapy effects in the solution

This section presents an end-to-end case study showcasing the functionality of TumorTwin. Throughout this section, we demonstrate the functionality of our package using the synthetic HGG dataset introduced in section “Example datasets provided with the package”. To illustrate the generalizability of the approach across different cancer types, we provide an analogous TNBC demonstration in Supplementary Material C. Figure 3 shows a representative forward simulation for the model described in the previous section, showing both the TTC (Eq. (9)) over time, and 2D snapshots from the full 3D solution domain at specific timepoints. We note that the model calibration case study shown here is idealized in the sense that there is no noise in the calibration data and fixed parameters are set to their ground-truth values. Prior work has investigated model calibration performance in more realistic settings, including pre-clinical models of glioma [27], clinical models of glioma [16], and clinical models of breast cancer [45]. The predictive capability of calibrated models, i.e., the extent to which calibrated predictions match future measurements, has also been investigated [17, 21, 59].

**Fig. 4:**
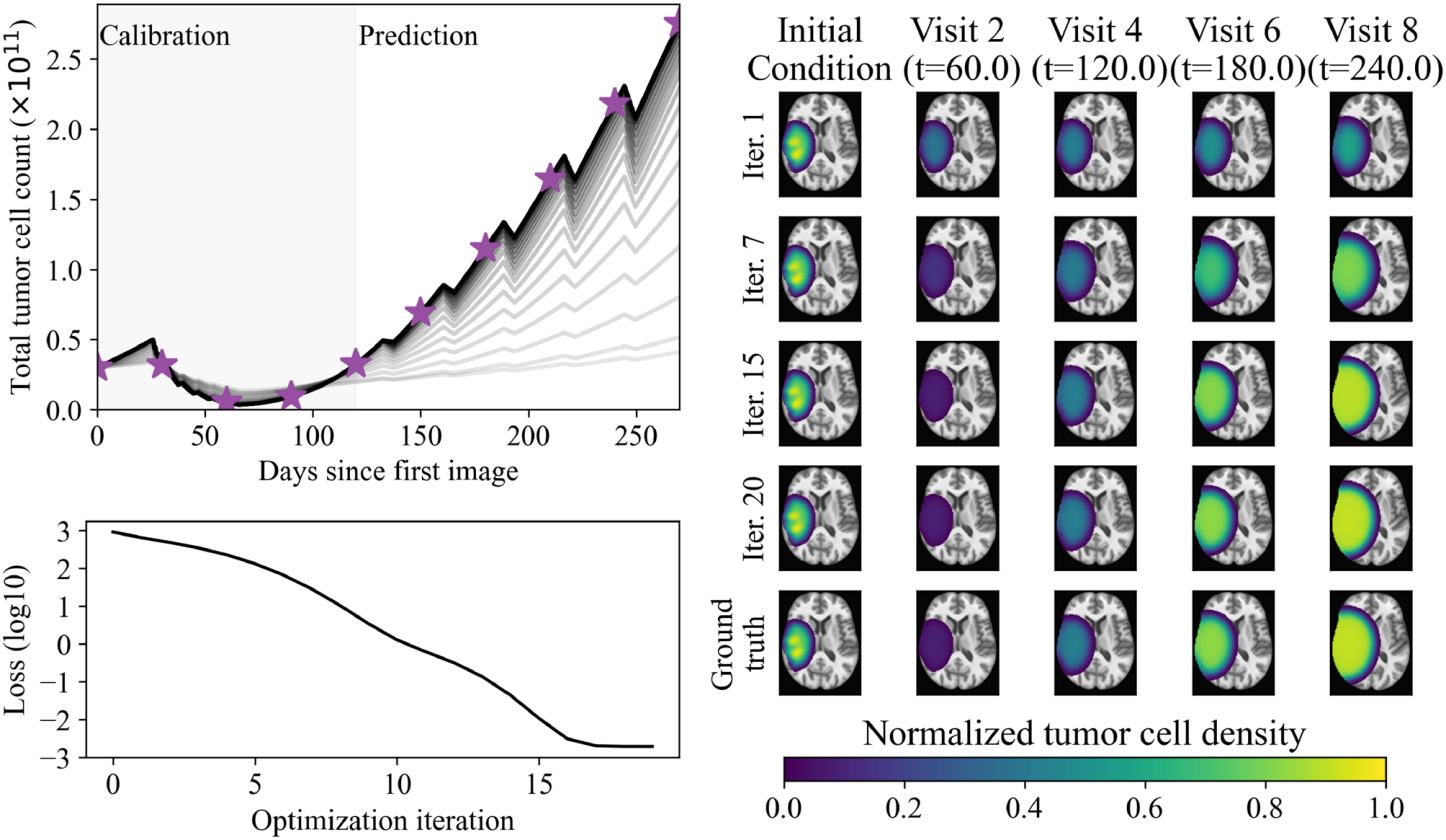
Model calibration to patient-specific MRI data. Top-left: total tumor cell count (TTC) time series for each iteration of the calibration (black lines; opacity increased with iteration) compared with the observed TTC (purple stars). Bottom-left: convergence of the loss function over optimization iterations. Right: evolution of a central tumor slice across time (left-to-right), and across optimization iterations (top-to-bottom). The bottom row shows the ground-truth data used for calibration and validation

To demonstrate the model calibration capability, we use the LM optimizer within PyTorch to calibrate a patient-specific DT using the in silico HGG dataset. For this example, we know the ground-truth model parameters used to generate the dataset (Fig. 3), but the optimizer is initiated with an initial guess of $k=0.01$, $D=0.02$, $\alpha_1 = 0.01$, $\alpha_\mathrm{RT}=0.04$ (20% of the respective truth values). Other parameters are fixed to their ground truth values. Figure 4 shows the results of running the optimizer with default optimizer parameter values. For this study, we calibrate the model to the first five imaging timepoints, while holding out the remaining images to assess predictive accuracy. We observe that the optimizer is able to calibrate the unknown model parameters to match the input MRI data within a few iterations, achieving between $10^{-4}$ and $10^{-6}$ relative error in parameter values. We also observe that the solver and optimizer are robust to large variations in the solution across iterations.

Here we have shown the predictive performance of the calibrated tumor growth model for a single treatment schedule. Note that, once calibrated, the model could be used to predict tumor growth and response to *alternative* treatment schedules, simply by changing the treatment parameters (dosage, timing) for treatments in the prediction regime. In this way, the calibrated model can form the basis of a patient-specific DT that could be used to issue predictions of future tumor growth, guide treatment decisions, and/or be re-calibrated whenever more MRI data are acquired.

### Empirical performance evaluation

**Fig. 5:**
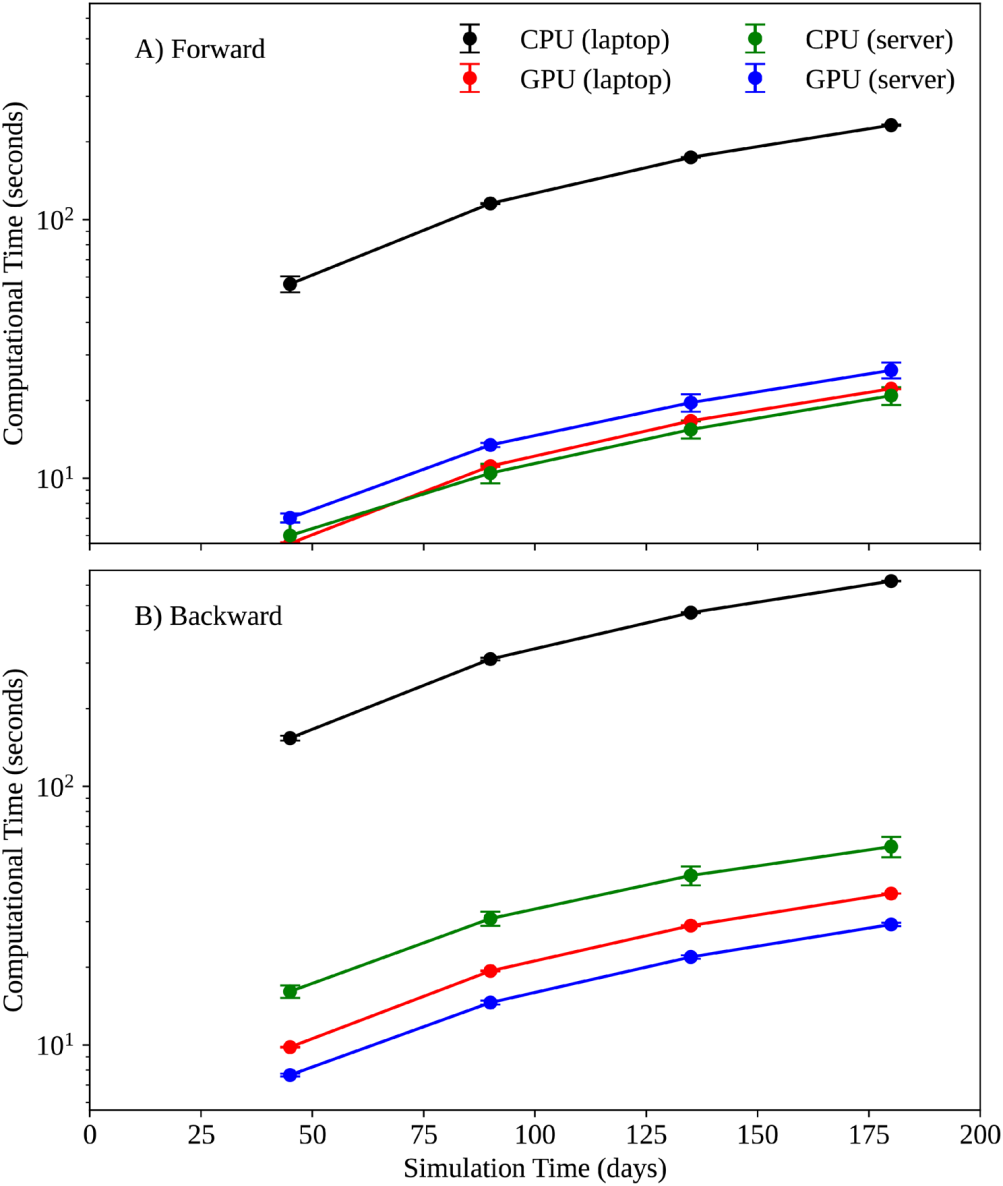
Solver performance profiling for CPU and GPU architectures. Top: mean and standard deviation of the wall-clock time required to compute a forward model prediction for 180 days. Bottom: results for a backward adjoint solve for the same period. Two CPU-based and two GPU-based hardware architectures were tested

Our focus in this work is on optimizing the computational performance of the model solver and calibration procedure (the latter scales with the former). We demonstrate the performance of our framework by comparing execution time on CPU vs. GPU (CUDA) architectures while varying the length of the tumor simulation between 45, 90, 135, and 180 days. The computational domain is a uniform 3D imaging grid of size ($166 \times 245 \times 48$) voxels, corresponding to a physical domain size of approximately (77.8 mm $\times$ 114.9 mm $\times$ 144.0 mm). This leads to a total of 1,952,160 degrees of freedom in the spatially discretized model. We solve the system of coupled ODEs given by Eq. (5), which governs tumor growth and treatment response dynamics. Figure 5 and Table 1 report wall-clock timings for forward simulations (45–180 days) and for the backward pass used to compute gradients during calibration.

**Table 1:** Comparison of solver execution time on CPU vs. GPU for forward (F) and backward (B) solver steps on a laptop and a server. The table shows the mean computational time (in seconds) for each solver step, along with the corresponding standard deviation. Speedup is calculated as the ratio of CPU to GPU time (values < indicate cases where the CPU is faster)

Solver Step	CPU Time (s)	GPU Time (s)	Speedup
Laptop			
F (45 days)	56.36 $\pm$ 3.99	5.60 $\pm$ 0.05	**10.07** $\pm$ **0.72**
F (90 days)	115.50 $\pm$ 0.58	11.14 $\pm$ 0.08	**10.37** $\pm$ **0.09**
F (135 days)	174.11 $\pm$ 0.56	16.66 $\pm$ 0.08	**10.45** $\pm$ **0.06**
F (180 days)	231.97 $\pm$ 1.15	22.19 $\pm$ 0.07	**10.45** $\pm$ **0.06**
B (45 days)	153.75 $\pm$ 3.34	9.81 $\pm$ 0.03	**15.67** $\pm$ **0.34**
B (90 days)	310.74 $\pm$ 3.60	19.34 $\pm$ 0.07	**16.07** $\pm$ **0.20**
B (135 days)	469.64 $\pm$ 1.63	28.95 $\pm$ 0.15	**16.22** $\pm$ **0.10**
B (180 days)	622.31 $\pm$ 1.98	38.51 $\pm$ 0.03	**16.16** $\pm$ **0.05**
Server			
F (45 days)	6.01 $\pm$ 0.75	7.02 $\pm$ 0.28	**0.86** $\pm$ **0.11**
F (90 days)	10.46 $\pm$ 0.91	13.45 $\pm$ 0.24	**0.78** $\pm$ **0.07**
F (135 days)	15.43 $\pm$ 1.19	19.61 $\pm$ 1.52	**0.79** $\pm$ **0.09**
F (180 days)	20.87 $\pm$ 1.66	26.16 $\pm$ 1.84	**0.80** $\pm$ **0.08**
B (45 days)	16.10 $\pm$ 0.88	7.66 $\pm$ 0.10	**2.10** $\pm$ **0.12**
B (90 days)	30.82 $\pm$ 1.95	14.59 $\pm$ 0.25	**2.11** $\pm$ **0.14**
B (135 days)	45.23 $\pm$ 3.78	21.90 $\pm$ 0.35	**2.07** $\pm$ **0.18**
B (180 days)	58.49 $\pm$ 5.28	29.26 $\pm$ 0.46	**2.00** $\pm$ **0.18**

**Profiling settings.** All timings use the torchdiffeqrk4 integrator with a fixed step size of 0.5 days (see Section “Importing and pre-processing patient data”). Backward-pass timings correspond to adjoint-based gradient computation using torchdiffeq with use_adjoint = True.

**Hardware.** All experiments were conducted on two different systems, representative of computational resources typically available to researchers: a Dell Inspiron 16 Plus 7630 laptop running Ubuntu 22.04.4 LTS, equipped with an Intel Core i7-13700 H processor (14 cores, 20 threads, 5.0 GHz max clock), 32 GB RAM, and an NVIDIA GeForce RTX 4060 Laptop GPU (8 GB VRAM, CUDA 12.2); and a Dell PowerEdge R740 server running Ubuntu 22.04.5 LTS, equipped with an Intel Xeon Gold 6248 R processor (48 cores, 96 threads, 4.0 GHz max clock), 187 GB RAM, and an NVIDIA A100 PCIe GPU (40 GB VRAM, CUDA 12.4).

Our results show that forward solves scale roughly linearly with the prediction length. The benefit of GPU acceleration depends on both hardware and pass type. On the laptop hardware, we observe an approximately $10\times$ speedup running on the GPU vs. CPU, while backward solves (gradient-based calibration) are more computationally expensive but benefit from an approximately $16\times$ GPU speedup. This acceleration is particularly important when performing deterministic calibration, where many iterations of forward and backward simulations may be required. On the server, forward solves are faster on the CPU than on the GPU for the tested simulation lengths (speedup $<$1), whereas the backward pass remains faster on the GPU (approximately $2\times$ speedup). These results highlight two regimes: GPUs substantially accelerate gradient computations (critical for calibration), while high-core-count server CPUs can be competitive or faster for forward simulations in this configuration. Overall, these results suggest that our framework enables the prediction of tumor growth over a period of one year in roughly one minute, with a backward pass in roughly $1-2$ minutes, on either a consumer grade GPU or a high-performance CPU.

## Discussion

While the technology underlying TumorTwin, namely image-guided predictive computational models of cancer growth and treatment, has shown transformative potential in oncology. There are numerous barriers to overcome before the technology is ready for real-world clinical application.

First, the effect of image and model resolution on prediction quality and computational cost is not well explored. In the results presented here, we set the model resolution equal to the image resolution, but in general these could be decoupled by resampling the image data onto a model grid of arbitrary resolution. Systematically exploring the relationship between resolution and computational cost will require running many solves with different model resolutions and image characterisics, which modular and high-performance implementations such as TumorTwin can greatly accelerate.

Second, there are several modeling choices and model extensions that could be explored by leveraging our modular approach to extend the functionality of TumorTwin. The first of these is the introduction of spatially varying model parameters. Homogenization of model parameters leads to a parsimonious model that can efficiently be optimized, but lacks the ability to faithfully capture the intricate intra-tumoral heterogeneity observed in the real world. High-dimensional parameters have been used by the authors and others to better describe the heterogeneous nature of the tumor and its microenvironment through, for example, spatially-varying proliferation rates [17], heterogeneous delivery of chemotherapy [24], tissue-specific diffusion coefficients [35, 53], and tissue mechanical properties [8]. However, introducing high-dimensional parameters increases computational overhead and will require further developments, including scalable methods and surrogates [9, 38], to enable analysis in clinically actionable timelines. Other model extensions that could be explored include distinguishing between enhancing (cell-dense tumor) and non-enhancing regions (infiltrative tumor and/or peritumoral edema) [17], as well as the multi-species model introduced by Hawkins-Daarud et al. [15], which explicitly incorporates edema dynamics. All of these extensions could be explored within the TumorTwin framework by implementing a new TumorGrowthModel within the framework.

Third, uncertainty quantification and subsequent optimization under uncertainty. Despite considerable uncertainties in the data collection process and data-driven estimation of tumor properties, the uncertainty quantification of tumor growth models is still in its infancy [6, 14, 32–34]. In addition to increased certifiability of model predictions, uncertainty quantification opens the door for robust decision-making through the minimization of tail risks [5, 6, 56]. This codebase establishes a high-performance foundation, which we plan to extend with rigorous uncertainty quantification to establish trust and accuracy and enable systemic model validation [36]. Since this codebase is built on PyTorch, any uncertainty quantification and propagation packages that leverage PyTorch’s native random variable framework should be directly applicable.

Fourth, a final avenue for future development involves the integration of machine learning techniques into the DT architecture. Since TumorTwin is tightly integrated with Pytorch, it can seamlessly integrate with other pytorch-based machine learning models. For example, a Pytorch-based convolutional neural network may be integrated as part of the MRI processing pipeline, or a neural network could be used to represent a spatially varying model parameter with gradient computation naturally extending to the network hyperparameters.

Finally, we emphasize that TumorTwin is intended only for research-use to explore the types of research questions outlined above. Developing this technology into a regulatory-grade clinical decision-support tool requires significant future effort on clinical validation (including large-scale, multi-institutional clinical studies), healthcare system integration, and model governance.

## Conclusion

This work introduces TumorTwin, an open-source Python framework for initializing and personalizing image-based DTs for oncology applications. The codebase is modular and adaptable, and represents a significant step towards a common framework for pan-cancer DTs. Many DT use-cases in oncology, for example, tumor response prediction [17], radiotherapy optimization [34], and chemotherapy optimization [58] are shared across disease sites, so success in one site can be rapidly translated and developed in other sites using this framework. By leveraging a modular architecture, researchers can easily integrate alternative data sources, tumor growth models, treatment models, and numerical algorithms, enabling efficient exploration of modeling choices and their impact on DT performance. This is supported by a complete and robust default architecture, with support for efficient gradient computation exploiting the differentiable programming framework and GPU acceleration available through PyTorch.

## Electronic supplementary material

Below is the link to the electronic supplementary material.

Supplementary Material 1

Supplementary Material 2

Supplementary Material 3

## Figures and Tables

**Fig. 6:**
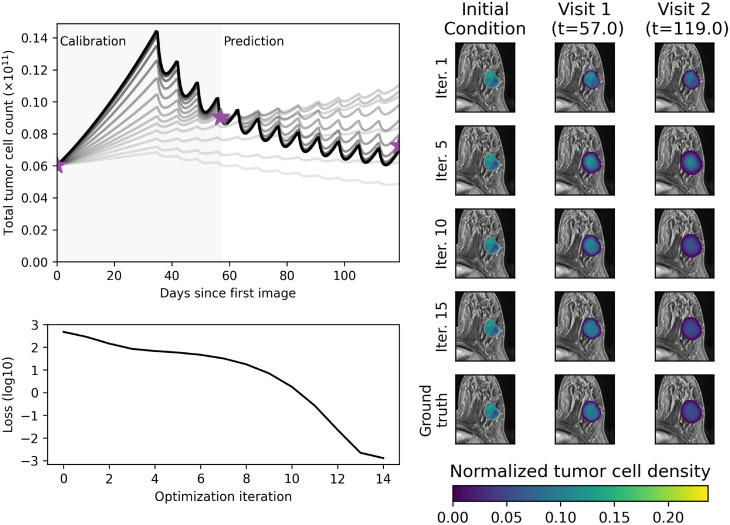
Model calibration to patient-specific MRI data - triple-negative breast cancer example. Top-left: total tumor cell count (TTC) time series for each iteration of the calibration (black lines; opacity increased with iteration) compared with the observed TTC (purple stars). Bottom-left: convergence of the loss function over optimization iterations. Right: evolution of a central tumor slice across time (left-to-right), and across optimization iterations (top-to-bottom). The bottom row shows the ground-truth data used for calibration and validation

## Data Availability

The demo data sets supporting the results of this article are available in the code repository under input_files. https://github.com/OncologyModelingGroup/TumorTwin. The original data used to develop the demo data is available at https://www.nitrc.org/projects/sri24 and https://doi.org/10.7937/TCIA.D8Z0-9T85.

## A Summary of model parameters and variables

**Table 2:** Summary of parameters and variables, along with their definition and units

Tumor growth and response parameters and variables		
Parameter or variable	Definition	Units
$N$	Normalized tumor cell density	unitless
$D$	Tumor cell diffusion coefficient	$mm^2/day$
$k$	Tumor cell proliferation rate	$day^-1$
$\theta$	Carrying Capacity	unitless
$n_{CT}$	Number of chemotherapy agents	unitless
$\alpha_i$	Chemotherapy efficacy for agent *i*	$day^-1$
$\beta_i$	Decay rate for chemotherapy agent *i*	$day^-1$
$\tau_{i,j}$	*j-th* administration time for agent *i*	$day$
$T_i$	Total number of doses for agent *i*	unitless
$\alpha_{RT}$	Radiotherapy sensitivity parameter (linear component)	$Gy^-1$
$\beta_{RT}$	Radiotherapy sensitivity parameter (quadratic component)	$Gy^-2$
$d_{RT}(t)$	Radiotherapy dose at time $t$	$Gy$
$\theta_{cells}$	Maximum number of tumor cells per voxel	cells
$TTC$	Total tumor cell count	cells
**Additional parameters and variables**		
**Parameter or variable**	**Definition**	**Units**
$ADC$	Apparent diffusion coefficient	$mm^2/s$
$ADC_W$	Apparent diffusion coefficient of water	$mm^2/s$
$ADC_{min}$	Minimum apparent diffusion coefficient	$mm^2/s$
$\textit{\textbf{p}}$	Set of mathematical model parameters	NA
$\textit{\textbf{p*}}$	Set of patient-specific model parameters	NA
$o(t_i)$	Observational data at time $t_i$	NA
$n_{visit}$	Number of imaging visits	NA
$n_{p}$	Number of patient-specific parameters	NA
$u_{0},N_0$	Initial condition	NA

## B Description of synthetic cases and treatment regimens

### B.1 Description of synthetic cases and treatment regimens

We created two synthetic datasets to demonstrate TumorTwin in two different disease sites and treatment paradigms. In particular, we consider HGG and TNBC, where both tests cases were synthesized using images from publicly available datasets. The HGG example provides synthetic longitudinal collected before,during and after RT, with the RT and CT regimens dictated by the current standard of care treatment protocol [51]. The treatment consists of RT (delivered in 2 Gy fractions over 30 sessions (5 days per week for 6 weeks), alongside daily oral temozolomide at 75 $\mathrm{mg}/\mathrm{m}^2$ for the entire radiotherapy period. Following a 4-week break, patients undergo 6 cycles of adjuvant CT, administered at 150–200 $\mathrm{mg}/\mathrm{m}^2$ per day for 5 days in each 28-day cycle. Likewise, the TNBC example provides synthetic longitudinal data collected before, during and after the delivery of neoadjuvent CT. In the synthetic case, we consider the weekly delivery of Paclitaxel administered at 80 $\mathrm{mg}/\mathrm{m}^2$ weekly for 12 weeks. In this section we detail the image processing and modeling techniques used to generate both synthetic datasets.

### B.2 Generation of synthetic longitudinal imaging studies

For the HGG case, we seeded an artificial tumor within the SRI24 normal adult brain atlas [47] and evolved it using Eqs. (3)-(4) with the following parameter values: $$k = 0.05, D = 0.1\ \mathrm{mm}^2/\mathrm{day},$$$$\alpha_\mathrm{RT} = 0.05\ \mathrm{Gy}^{-1}, \beta_\mathrm{RT} = 0.005\ \mathrm{Gy}^{-2},$$$$\alpha_1 = 0.2\ \mathrm{day}^{-1}$$, and $$\beta_1 = 9.242\ \mathrm{day}^{-1}$$ [42]. This atlas also provided $$T_1$$ and $$T_2$$ weighted images which we used to define the brain mask. The synthetic HGG growth and response was sampled every 45 days until day 225 and the numerical time step was assigned to $$\delta t = 0.1\ \mathrm{day}^{-1}$$.

For the TNBC data we used anatomical ($T_1$-weighted pre- and post-contrast) and functional ($ADC$) imaging data from case 104,268 from the Investigation of Serial studies to Predict Your Therapeutic Response with Imaging And molecular analysis 2 (I-SPY 2) dataset [31, 41]. All images were registered to the $T_1$-weighted MRI using a rigid registration using a rigid registration using imregtform in MATLAB R2024b [23]. We then used the radiologist drawn tumor segmentation included within the I-SPY2 dataset. A breast mask was created by a manual intensity threshold followed by filling holes using imfill in MATLAB. Normalized tumor cell density maps were generated using Eq. (7). Using the pre-treatment visit, we then simulated TNBC growth using the following parameters: $D = 0.1\ \mathrm{mm}^2/\mathrm{day}$, $k = 0.05$, $\alpha_\mathrm{RT} = 0\ \mathrm{Gy}^{-1}$, $\beta_\mathrm{RT} = 0\ \mathrm{Gy}^{-2}$, $\alpha = 0.2\ \mathrm{day}^{-1}$, and $\beta = 0.7\ \mathrm{day}^{-1}$ [42]. The synthetic TNBC growth and response was sampled at the time points available for the real I-SPY dataset.

In both cases, NIfTI-formatted images of the tumor segmentations are generated at each visit by applying a threshold of $N(\boldsymbol{x},t) > 0.001$. We then used the segmentations, $N(\boldsymbol{x},t)$, and Eq. (7) to calculate the $ADC$ for each corresponding $N(\boldsymbol{x},t)$ map which is saved as NIfTI-formatted images. Lastly, we create unique JSON patient configuration files detailing the treatment and imaging schedule for each synthetic case.

## C Model prediction and calibration results for triple-negative breast cancer

To demonstrate the applicability of the TumorTwin framework to different cancer sites, we here present calibration results for the in-silico TNBC dataset described in the Methods section. Figure 6 presents these results, and is analogous to the calibration results for HGG provided in 4. Here we again use the LM optimizer to calibrate a patient-specific DT model to the first two visits of MRI data, leaving the third visit out to assess predictive capability. For this example, we know the ground-truth model parameters (used to generate the dataset), but the optimizer is initiated with an initial guess of $k=0.005$, $D=0.01$, and $\alpha_1=0.04$ (20% of the truth values). Other parameters are fixed to their ground truth values. The optimizer parameters are the same default values as used for the HGG demonstration. We again observe that the optimizer is able to calibrate the unknown model parameters to match the input MRI data within a few iterations, and is again robust to large variations in the solution across iterations.

## Abbreviations

CPU: Central processing unit
CT: Chemotherapy
DT: Digital twin
GPU: Graphical processing unit
HGG: High grade glioma
JSON: JavaScript object notation
MRI: Magnetic resonance imaging
NIfTI: Neuroimaging informatics technology initiative
ODE: Ordinary differential equations
RT: Radiotherapy
TNBC: Triple-negative breast cancer

## Code availability

Project name: TumorTwin. Project home page: TumorTwin GitHub. Operating system(s): Platform independent. Programming language: Python (version: “> = 3.9, <3.12”). Other requirements: TumorTwin TOML. License: TumorTwin License. Any restrictions to use by non-academics: Commercial use restrictions - see license file provided with code for full terms.
