# Supplementary material for: TumorTwin: a Python framework for patient-specific digital twins in oncology
Source: BMC Med Inform Decis Mak. 2026 May 11;26:237. doi: 10.1186/s12911-026-03520-2 (PMC13330372; doi:10.1186/s12911-026-03520-2)
Supplement: Supplementary file 1 — Supplementary Material 1 [file 12911_2026_3520_MOESM1_ESM.pdf]

A Summary of model parameters and variables

Table 2: Parameters and variables

Tumor growth and response parameters and variables		
**Parameter or variable**	**Definition**	**Units**
N	Normalized tumor cell density	unitless
D	Tumor cell diffusion coefficient	mm^2/day
k	Tumor cell proliferation rate	day^-1
θ	Carrying Capacity	unitless
n_{CT}	Number of chemotherapy agents	unitless
α_i	Chemotherapy efficacy for agent i	day^-1
β_i	Decay rate for chemotherapy agent i	day^-1
$\tau_{i,j}$	*j-th* administration time for agent i	day
T_i	Total number of doses for agent i	unitless
α_{RT}	Radiotherapy sensitivity parameter (linear component)	Gy^-1
β_{RT}	Radiotherapy sensitivity parameter (quadratic component)	Gy^-2
$d_{RT}(t)$	Radiotherapy dose at time t	Gy
θ_{cells}	Maximum number of tumor cells per voxel	cells
TTC	Total tumor cell count	cells
**Additional parameters and variables**		
**Parameter or variable**	**Definition**	**Units**
ADC	Apparent diffusion coefficient	mm^2/s
ADC_W	Apparent diffusion coefficient of water	mm^2/s
ADC_{min}	Minimum apparent diffusion coefficient	mm^2/s
$\boldsymbol{p}$	Set of mathematical model parameters	NA
$\boldsymbol{p*}$	Set of patient-specific model parameters	NA
$o(t_i)$	Observational data at time t_i	NA
n_{visit}	Number of imaging visits	NA
n_p	Number of patient-specific parameters	NA
u_0, N_0	Initial condition	NA
