# Supplementary material for: TumorTwin: a Python framework for patient-specific digital twins in oncology
Source: BMC Med Inform Decis Mak. 2026 May 11;26:237. doi: 10.1186/s12911-026-03520-2 (PMC13330372; doi:10.1186/s12911-026-03520-2)
Supplement: Supplementary file 2 — Supplementary Material 2 [file 12911_2026_3520_MOESM2_ESM.pdf]

B Description of Synthetic Cases and Treatment Regimens

B.1 Description of Synthetic Cases and Treatment Regimens

We created two synthetic datasets to demonstrate `TumorTwin` in two different disease sites and treatment paradigms. In particular, we consider HGG and TNBC, where both tests cases were synthesized using images from publicly available datasets. The HGG example provides synthetic longitudinal collected before,during and after RT, with the RT and CT regimens dictated by the current standard of care treatment protocol [51]. The treatment consists of RT (delivered in 2 Gy fractions over 30 sessions (5 days per week for 6 weeks), alongside daily oral temozolomide at 75 mg/m^2 for the entire radiotherapy period. Following a 4-week break, patients undergo 6 cycles of adjuvant CT, administered at 150–200 mg/m^2 per day for 5 days in each 28-day cycle. Likewise, the TNBC example provides synthetic longitudinal data collected before, during and after the delivery of neoadjuvent CT. In the synthetic case, we consider the weekly delivery of Paclitaxel administered at 80 mg/m^2 weekly for 12 weeks. In this section we detail the image processing and modeling techniques used to generate both synthetic datasets.

B.2 Generation of synthetic longitudinal imaging studies

For the HGG case, we seeded an artificial tumor within the SRI24 normal adult brain atlas [47] and evolved it using Eqs. (3)-(4) with the following parameter values: $k = 0.05, D = 0.1$ mm^2/day, $\alpha_{\text{RT}} = 0.05$ Gy^{-1}, $\beta_{\text{RT}} = 0.005$ Gy^{-2}, $\alpha_1 = 0.2$ day^{-1}, and $\beta_1 = 9.242$ day^{-1} [42]. This atlas also provided T_1 and T_2 weighted images which we used to define the brain mask. The synthetic HGG growth and response was sampled every 45 days until day 225 and the numerical time step was assigned to $\delta t = 0.1$ day^{-1}.

For the TNBC data we used anatomical (T_1-weighted pre- and post-contrast) and functional (ADC) imaging data from case 104268 from the Investigation of Serial studies to Predict Your Therapeutic Response with Imaging And molecular analysis 2 (I-SPY 2) dataset [41, 31]. All images were registered to the T_1-weighted MRI using a rigid registration using a rigid registration using `imregtform` in MATLAB R2024b [23]. We then used the radiologist drawn tumor segmentation included within the I-SPY2 dataset. A breast mask was created by a manual intensity threshold followed by filling holes using `imfill` in MATLAB. Normalized tumor cell density maps were generated using Equation (7). Using the pre-treatment visit, we then simulated TNBC growth using the following parameters: $D = 0.1$ mm^2/day, $k = 0.05$, $\alpha_{\text{RT}} = 0$ Gy^{-1}, $\beta_{\text{RT}} = 0$ Gy^{-2}, $\alpha = 0.2$ day^{-1}, and $\beta = 0.7$ day^{-1} [42]. The synthetic TNBC growth and response was sampled at the time points available for the real I-SPY dataset.

In both cases, NIfTI-formatted images of the tumor segmentations are generated at each visit by applying a threshold of $N(\boldsymbol{x}, t) > 0.001$. We then used the segmentations, $N(\boldsymbol{x}, t)$, and Equation (7) to calculate the ADC for each corresponding $N(\boldsymbol{x}, t)$ map which is saved as NIfTI-formatted images. Lastly, we create unique JSON patient configuration files detailing the treatment and imaging schedule for each synthetic case.

References

[1] A. Abbasi, S. Amjad-Iranagh, and B. Dabir. Cellsys: An open-source tool for building initial structures for bio-membranes and drug-delivery systems. *Journal of Computational Chemistry*, 43(5):331–339, 2022.

[2] A. W. Anderson, J. Xie, J. Pizzonia, R. A. Bronen, D. D. Spencer, and J. C. Gore. Effects of cell volume fraction changes on apparent diffusion in human cells. *Magnetic Resonance Imaging*, 18(6):689–695, 2000.

[3] S. Bakas, M. Reyes, A. Jakab, S. Bauer, M. Rempfler, A. Crimi, R. T. Shinohara, C. Berger, S. M. Ha, M. Rozycki, et al. Identifying the best machine learning algorithms for brain tumor segmentation, progression assessment, and overall survival prediction in the brats challenge. *arXiv preprint arXiv:1811.02629*, 2018.

[4] R. R. Bravo, E. Baratchart, J. West, R. O. Schenck, A. K. Miller, J. Gallaher, C. D. Gatenbee, D. Basanta, M. Robertson-Tessi, and A. R. Anderson. Hybrid automata library: A flexible platform for hybrid modeling with real-time visualization. *PLoS computational biology*, 16(3):e1007635, 2020.

[5] A. Chaudhuri, B. Kramer, M. Norton, J. O. Royset, and K. Willcox. Certifiable risk-based engineering design optimization. *AIAA Journal*, 60(2):551–565, 2022.

[6] A. Chaudhuri, G. Pash, D. A. Hormuth II, G. Lorenzo, M. Kapteyn, C. Wu, E. A. B. F. Lima, T. E. Yankeelov, and K. Willcox. Predictive Digital Twin for Optimizing Patient-Specific Radiotherapy Regimens under Uncertainty in High-Grade Gliomas. *Frontiers in Artificial Intelligence*, 6, 2023.

[7] R. T. Q. Chen. torchdiffeq, 2018. https://github.com/rtqichen/torchdiffeq.

[8] X. Chen, R. M. Summers, and J. Yao. Kidney tumor growth prediction by coupling reaction–diffusion and biomechanical model. *IEEE Transactions on Biomedical Engineering*, 60(1):169–173, 2013.

[9] C. Christenson, C. Wu, D. A. Hormuth II, C. E. Stowers, M. LaMonica, J. Ma, G. M. Rauch, and T. E. Yankeelov. Fast model calibration for predicting the response of breast cancer to chemotherapy using proper orthogonal decomposition. *Journal of Computational Science*, 82:102400, 2024.

[10] J. R. Dormand and P. J. Prince. A family of embedded runge-kutta formulae. *Journal of computational and applied mathematics*, 6(1):19–26, 1980.

[11] A. Ghaffarizadeh, R. Heiland, S. H. Friedman, S. M. Mumenthaler, and P. Macklin. Physicell: An open source physics-based cell simulator for 3-d multicellular systems. *PLoS computational biology*, 14(2):e1005991, 2018.

[12] O. Ghattas and K. Willcox. Learning physics-based models from data: perspectives from inverse problems and model reduction. *Acta Numerica*, 30:445–554, 2021.

[13] E. Hairer, S. P. Nørsett, and G. Wanner. *Solving ordinary differential equations I (2nd revised. ed.): nonstiff problems*. Springer-Verlag, Berlin, Heidelberg, 1993.

[14] A. Hawkins-Daarud, S. Prudhomme, K. G. van der Zee, and J. T. Oden. Bayesian calibration, validation, and uncertainty quantification of diffuse interface models of tumor growth. *Journal of Mathematical Biology*, 67(6):1457–1485, 2013.

[15] A. Hawkins-Daarud, R. C. Rockne, A. R. A. Anderson, and K. R. Swanson. Modeling tumor-associated edema in gliomas during anti-angiogenic therapy and its impact on imageable tumor. *Frontiers in Oncology*, 3:66, 2013.

[16] K. C. Hiremath, K. Atakishi, E. A. Lima, M. Farhat, B. Panthi, H. Langshaw, M. D. Shanker, W. Talpur, S. Thrower, J. Goldman, et al. Identifiability and model selection frameworks for models of high-grade glioma response to chemoradiation. *Philosophical Transactions A*, 383(2293):20240212, 2025.

[17] D. A. Hormuth, K. A. Al Feghali, A. M. Elliott, T. E. Yankeelov, and C. Chung. Image-based personalization of computational models for predicting response of high-grade glioma to chemoradiation. *Scientific reports*, 11(1):8520, 2021.

[18] D. A. Hormuth, M. Farhat, C. Christenson, B. Curl, C. Chad Quarles, C. Chung, and T. E. Yankeelov. Opportunities for improving brain cancer treatment outcomes through imaging-based mathematical modeling of the delivery of radiotherapy and immunotherapy. *Advanced Drug Delivery Reviews*, 187:114367, 2022. Publisher: Elsevier B.V.

[19] D. A. Hormuth, A. M. Jarrett, and T. E. Yankeelov. Forecasting tumor and vasculature response dynamics to radiation therapy via image based mathematical modeling. *Radiation Oncology*, 15:1–14, 2020.

[20] D. A. Hormuth, J. A. Weis, S. L. Barnes, M. I. Miga, E. C. Rericha, V. Quaranta, and T. E. Yankeelov. A mechanically coupled reaction–diffusion model that incorporates intra-tumoural heterogeneity to predict in vivo glioma growth. *Journal of The Royal Society Interface*, 14(127):20161010, 2017.

[21] D. A. Hormuth, II, M. Farhat, B. Panthi, H. Langshaw, M. D. Shanker, W. Talpur, S. Thrower, J. Goldman, S. Ty, C. Custer, J. Kowalski, T. E. Yankeelov, and C. Chung. Forecasting chemoradiation response midtreatment for high-grade gliomas through patient-specific biology-based modeling. *International Journal of Radiation Oncology*Biology*Physics*, 123(5):1413–1427, dec 2025. Epub 2025 Jul 25.

[22] D. A. Hormuth II, K. A. A. Feghali, A. M. Elliott, T. Yankeelov, and C. Chung. Image-based personalization of computational models for predicting response of high-grade glioma to chemoradiation. *Scientific Reports*, 11:1–14, 2021. Publisher: Nature Publishing Group UK ISBN: 4159802187887.

[23] T. M. Inc. MATLAB version: R2024b, 2024.

[24] A. M. Jarrett, A. S. Kazerouni, C. Wu, J. Virostko, A. G. Sorace, J. C. DiCarlo, D. A. Hormuth, D. A. Ekrut, D. Patt, B. Goodgame, S. Avery, and T. E. Yankeelov. Quantitative magnetic resonance imaging and tumor forecasting of breast cancer patients in the community setting. *Nature Protocols*, 16(11):5309–5338, 2021.

[25] P. Kidger, R. T. Q. Chen, and T. J. Lyons. "hey, that's not an ode": Faster ode adjoints via seminorms. *International Conference on Machine Learning*, 2021.

[26] A. R. Kutuva, J. J. Caudell, K. Yamoah, H. Enderling, and M. U. Zahid. Mathematical modeling of radiotherapy: impact of model selection on estimating minimum radiation dose for tumor control. *Frontiers in Oncology*, Volume 13 - 2023, 2023.

[27] M. F. LaMonica, T. E. Yankeelov, and D. A. Hormuth II. Investigating the limits of predictability of magnetic resonance imaging-based mathematical models of tumor growth. *Cancers*, 17(20), 2025.

[28] R. Laubenbacher, B. Mehrad, I. Shmulevich, and N. Trayanova. Digital twins in medicine. *Nature Computational Science*, 4(3):184–191, 2024.

[29] K. Leder, K. Pitter, Q. LaPlant, D. Hambardzumyan, B. D. Ross, T. A. Chan, E. C. Holland, and F. Michor. Mathematical modeling of pdgf-driven glioblastoma reveals optimized radiation dosing schedules. *Cell*, 156(3):603–616, 2014.

[30] R. J. LeVeque. *Finite difference methods for ordinary and partial differential equations: steady-state and time-dependent problems.* SIAM, 2007.

[31] W. Li, D. C. Newitt, J. Gibbs, L. J. Wilmes, E. F. Jones, V. A. Arasu, F. Strand, N. Onishi, A. A.-T. Nguyen, J. Kornak, B. N. Joe, E. R. Price, H. Ojeda-Fournier, M. Eghtedari, K. W. Zamora, S. A. Woodard, H. Umphrey, W. Bernreuter, M. Nelson, and N. M. Hylton. I-spy 2 breast dynamic contrast enhanced mri trial (ispy2) (version 1) [data set], 2022. Accessed: 2025-03-20.

[32] B. Liang, J. Tan, L. Lozenski, D. A. Hormuth, T. E. Yankeelov, U. Villa, and D. Faghihi. Bayesian inference of tissue heterogeneity for individualized prediction of glioma growth. *IEEE Transactions on Medical Imaging*, 42(10):2865–2875, 2023.

[33] E. Lima, J. Oden, B. Wohlmuth, A. Shahmoradi, D. Hormuth II, T. Yankeelov, L. Scarabosio, and T. Horger. Selection and validation of predictive models of radiation effects on tumor growth based on noninvasive imaging data. *Computer methods in applied mechanics and engineering*, 327:277–305, 2017.

[34] J. Lipkova, P. Angelikopoulos, S. Wu, E. Alberts, B. Wiestler, C. Diehl, C. Preibisch, T. Pyka, S. E. Combs, P. Hadjidoukas, K. Van Leemput, P. Koumoutsakos, J. Lowengrub, and B. Menze. Personalized radiotherapy design for glioblastoma: Integrating mathematical tumor models, multimodal scans, and bayesian inference. *IEEE Transactions on Medical Imaging*, 38(8):1875–1884, August 2019.

[35] J. Lipková, B. Menze, B. Wiestler, P. Koumoutsakos, and J. S. Lowengrub. Modelling glioma progression, mass effect and intracranial pressure in patient anatomy. *Journal of the Royal Society Interface*, 19(188):20210922, 2022.

[36] G. Lorenzo, D. A. Hormuth II, C. Wu, G. Pash, A. Chaudhuri, E. A. Lima, L. C. Okereke, R. Patel, K. Willcox, and T. E. Yankeelov. Validating the predictions of mathematical models describing tumor growth and treatment response. *arXiv preprint arXiv:2502.19333*, 2025.

[37] S. J. McMahon. The linear quadratic model: usage, interpretation and challenges. *Physics in Medicine & Biology*, 64(1):01TR01, 2018.

[38] J. Metzcar, C. R. Jutzeler, P. Macklin, A. Köhn-Luque, and S. C. Brüningk. A review of mechanistic learning in mathematical oncology. *Frontiers in Immunology*, 15, 2024.

[39] G. R. Mirams, C. J. Arthurs, M. O. Bernabeu, R. Bordas, J. Cooper, A. Corrias, Y. Davit, S.-J. Dunn, A. G. Fletcher, D. G. Harvey, et al. Chaste: an open source c++ library for computational physiology and biology. *PLoS computational biology*, 9(3):e1002970, 2013.

[40] National Academy of Engineering and E. National Academies of Sciences, and Medicine. *Foundational Research Gaps and Future Directions for Digital Twins*. The National Academies Press, Washington, DC, 2023.

[41] D. C. Newitt, S. C. Partridge, Z. Zhang, J. Gibbs, T. Chenevert, M. Rosen, P. Bolan, H. Marques, J. Romanoff, L. Cimino, B. N. Joe, H. Umphrey, H. Ojeda-Fournier, B. Dogan, K. Y. Oh, H. Abe, J. Drukteinis, L. J. Esserman, and N. M. Hylton. Acrin 6698/i-spy2 breast dwi [data set], 2021. Accessed: 2025-03-20.

[42] W. Newman, J. Verweij, H. Rosing, K. Grunberg, S. Chattopadhyay, E. Gamelin, J. Klastersky, and E. K. Rowinsky. Pharmacokinetics of temozolomide: an oral cytotoxic agent with activity in the central nervous system. *Clinical Cancer Research*, 2(8):1105–1111, 1996.

[43] A. Niarakis, R. Laubenbacher, G. An, Y. Ilan, J. Fisher, Å. Flobak, K. Reiche, M. Rodríguez Martínez, L. Geris, L. Ladeira, et al. Immune digital twins for complex human pathologies: applications, limitations, and challenges. *NPJ systems biology and applications*, 10(1):141, 2024.

[44] G. Nocera, F. Sanvito, J. Yao, S. Oshima, S. A. Bobholz, et al. Independent histological validation of mr-derived radio-pathomic maps of tumor cell density using image-guided biopsies in human brain tumors. *Journal of Neuro-Oncology*, 175(1):111–122, 2025.

[45] C. M. Phillips, E. A. B. F. Lima, C. Wu, A. M. Jarrett, Z. Zhou, N. Elshafeey, J. Ma, G. M. Rauch, and T. E. Yankeelov. Assessing the identifiability of model selection frameworks for the prediction of patient outcomes in the clinical breast cancer setting. *Journal of Computational Science*, 69:102006, may 2023. Epub 2023 Apr 5.

[46] A. Quarteroni and A. Valli. *Numerical approximation of partial differential equations*, volume 23. Springer Science & Business Media, 2008.

[47] T. Rohlfing, N. M. Zahr, E. V. Sullivan, and A. Pfefferbaum. The sri24 multichannel atlas of normal adult human brain structure. *Human Brain Mapping*, 31(5):798–819, 2010.

[48] K. Sel, A. Hawkins-Daarud, A. Chaudhuri, D. Osman, A. Bahai, D. Paydarfar, K. Willcox, C. Chung, and R. Jafari. Survey and perspective on verification, validation, and uncertainty quantification of digital twins for precision medicine. *npj Digital Medicine*, 8(1):40, 2025.

[49] E. A. Stahlberg, M. Abdel-Rahman, B. Aguilar, A. Asadpoure, R. A. Beckman, L. L. Borkon, J. N. Bryan, C. M. Cebulla, Y. H. Chang, A. Chatterjee, J. Deng, S. Dolatshahi, O. Gevaert, E. J. Greenspan, W. Hao, T. Hernandez-Boussard, P. R. Jackson, M. Kuijjer, A. Lee, P. Macklin, S. Madhavan, M. D. McCoy, N. Mohammad Mirzaei, T. Razzaghi, H. L. Rocha, L. Shahriyari, I. Shmulevich, D. G. Stover, Y. Sun, T. Syeda-Mahmood, J. Wang, Q. Wang, and I. Zervantonakis. Exploring approaches for predictive cancer patient digital twins: Opportunities for collaboration and innovation. *Frontiers in Digital Health*, 4, 2022.

[50] J. Starruß, W. De Back, L. Brusch, and A. Deutsch. Morpheus: a user-friendly modeling environment for multiscale and multicellular systems biology. *Bioinformatics*, 30(9):1331–1332, 2014.

[51] R. Stupp, W. P. Mason, M. J. van den Bent, M. Weller, B. Fisher, M. J. Taphoorn, K. Belanger, A. A. Brandes, C. Marosi, U. Bogdahn, J. Curschmann, R. C. Janzer, S. K. Ludwin, T. Gorlia, A. Allgeier, D. Lacombe, G. Cairncross, E. Eisenhauer, and R. O. Mirimanoff. Radiotherapy plus concomitant and adjuvant temozolomide for glioblastoma. *New England Journal of Medicine*, 352(10):987–996, 2005.

[52] T. Sugahara, Y. Korogi, M. Kochi, I. Ikushima, Y. Shigematu, T. Hirai, T. Okuda, L. Liang, Y. Ge, Y. Komohara, Y. Ushio, and M. Takahashi. Usefulness of diffusion-weighted mri with echo-planar technique in the evaluation of cellularity in gliomas. *Journal of Magnetic Resonance Imaging*, 9(1):53–60, 1999.

[53] A. Swan, T. Hillen, J. C. Bowman, and A. D. Murtha. A patient-specific anisotropic diffusion model for brain tumour spread. *Bulletin of Mathematical Biology*, 80(5):1259–1291, May 2018.

[54] M. H. Swat, G. L. Thomas, J. M. Belmonte, A. Shirinifard, D. Hmeljak, and J. A. Glazier. Multi-scale modeling of tissues using compucell3d. In *Methods in cell biology*, volume 110, pages 325–366. Elsevier, 2012.

[55] S. Tisue and U. Wilensky. Netlogo: A simple environment for modeling complexity. In *International conference on complex systems*, volume 21, pages 16–21. Citeseer, 2004.

[56] R. Tyrrell Rockafellar and J. O. Royset. Engineering decisions under risk averseness. *ASCE-ASME Journal of Risk and Uncertainty in Engineering Systems, Part A: Civil Engineering*, 1(2):04015003, 2015.

[57] R. Woodhams, S. Ramadan, P. Stanwell, S. Sakamoto, H. Hata, M. Ozaki, S. Kan, and Y. Inoue. Diffusion-weighted imaging of the breast: principles and clinical applications. *Radiographics*, 31(4):1059–1084, 2011.

[58] C. Wu, D. A. Hormuth, G. Lorenzo, A. M. Jarrett, F. Pineda, F. M. Howard, G. S. Karczmar, and T. E. Yankeelov. Towards patient-specific optimization of neoadjuvant treatment protocols for breast cancer based on image-guided fluid dynamics. *IEEE Transactions on Biomedical Engineering*, 69(11):3334–3344, November 2022.

[59] C. Wu, A. M. Jarrett, Z. Zhou, N. Elshafeey, B. E. Adrada, R. P. Candelaria, R. M. M. Mohamed, M. Boge, L. Huo, J. B. White, D. Tripathy, V. Valero, J. K. Litton, C. Yam, J. B. Son, J. Ma, G. M. Rauch, and T. E. Yankeelov. MRI-based digital models forecast patient-specific treatment responses to neoadjuvant chemotherapy in triple-negative breast cancer. *Cancer Research*, 82(18):3394–3404, sep 2022.

[60] C. Wu, G. Lorenzo, D. A. Hormuth, E. A. B. F. Lima, K. P. Slavkova, J. C. DiCarlo, J. Virostko, C. M. Phillips, D. Patt, C. Chung, and T. E. Yankeelov. Integrating mechanism-based modeling with biomedical imaging to build practical digital twins for clinical oncology. *Biophysics Reviews*, 3(2):21304, May 2022. Publisher: American Institute of Physics.

[61] M. Zahid, A. Mohamed, J. Caudell, L. Harrison, C. Fuller, E. Moros, and H. Enderling. Dynamics-adapted radiotherapy dose (dard) for head and neck cancer radiotherapy dose personalization. *Journal of Personalized Medicine*, 11:1124, 2021.
