# Supplementary material for: TumorTwin: a Python framework for patient-specific digital twins in oncology
Source: BMC Med Inform Decis Mak. 2026 May 11;26:237. doi: 10.1186/s12911-026-03520-2 (PMC13330372; doi:10.1186/s12911-026-03520-2)
Supplement: Supplementary file 3 — Supplementary Material 3 [file 12911_2026_3520_MOESM3_ESM.pdf]

C Model prediction and calibration results for triple-negative breast cancer

To demonstrate the applicability of the `TumorTwin` framework to different cancer sites, we here present calibration results for the *in-silico* TNBC dataset described in the Methods section. Figure 6 presents these results, and is analogous to the calibration results for HGG provided in 4. Here we again use the LM optimizer to calibrate a patient-specific DT model to the first two visits of MRI data, leaving the third visit out to assess predictive capability. For this example, we know the ground-truth model parameters (used to generate the dataset), but the optimizer is initiated with an initial guess of $k = 0.005$, $D = 0.01$, and $\alpha_1 = 0.04$ (20% of the truth values). Other parameters are fixed to their ground truth values. The optimizer parameters are the same default values as used for the HGG demonstration. We again observe that the optimizer is able to calibrate the unknown model parameters to match the input MRI data within a few iterations, and is again robust to large variations in the solution across iterations.

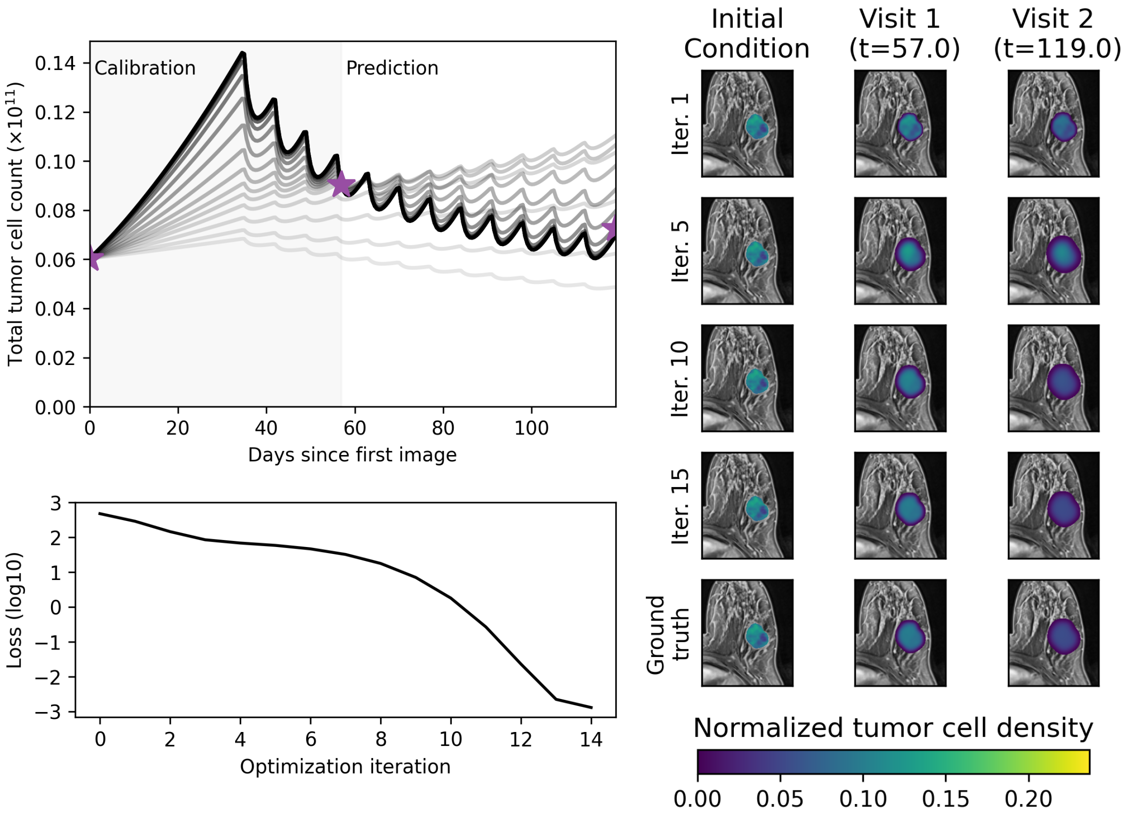

Figure 6: *Model calibration to patient-specific MRI data - Triple-negative breast cancer example.* Top-left: Total tumor cell count (TTC) time series for each iteration of the calibration (black lines; opacity increased with iteration) compared with the observed TTC (purple stars). Bottom-left: Convergence of the loss function over optimization iterations. Right: Evolution of a central tumor slice across time (left-to-right), and across optimization iterations (top-to-bottom). The bottom row shows the ground-truth data used for calibration and validation.
